# A Puff

**Published:** 1869-07

**Authors:** 


### A PUFF.

With a strong feeling of local pride we announce a recent discovery that our city is ahead of all competitors, in the way of professional progress. Other cities, too, may boast of colleges, journals, authors and other such small fry, but we have a regular "Dentestery!" But don't suppose that a man who can invent and RUN a "Dentestery" will cramp his genius by a single department of business, for the neatly painted sign adds "Kupping," and if that doesn't spell cupping what does it spell? Then follows "Leaching," which is followed by "Vaccination," and the "railroad speed" of the street car carried us beyond reading distance before we got half way down the sign-board; but we are bound to find out what that fellow does at his "Dentestery." W.

